# Chronic Tinnitus following Electroconvulsive Therapy

**DOI:** 10.1155/2011/607061

**Published:** 2011-09-18

**Authors:** Robert L. Folmer, Yongbing Shi, Sarah Theodoroff

**Affiliations:** ^1^National Center for Rehabilitative Auditory Research, Portland VA Medical Center, Portland, OR 97239, USA; ^2^Department of Otolaryngology, Oregon Health & Science University, Portland, OR 97239, USA

## Abstract

A 43-year-old female with a 27-year history of obsessive-compulsive disorder and major depression had previously been treated with psychotherapy, antidepressant, and antipsychotic medications. Because these treatments were minimally effective and because the frequency and duration of her depressive episodes continued to increase, the patient was scheduled to undergo a series of electroconvulsive therapy (ECT) procedures. The patient received four ECT treatments during one month. Stimulating current was delivered to the right frontotemporal region of the head. Electroencephalographic seizures occurred during each of the ECT procedures. After the patient recovered from anesthesia, she complained of headaches, muscle pain, amnesia, and, after the fourth ECT, she reported a ringing sound in her right ear. Audiometric testing the day after the fourth ECT revealed a slight increase in threshold for 8000 Hz tones in her right ear. It is likely that current delivered during the fourth ECT treatment triggered the perception of tinnitus for this patient. The unique organization of this patient's central nervous and auditory systems combined with her particular pharmacological history might have predisposed her to developing tinnitus.

## 1. Introduction

Chronic tinnitus is experienced by millions of people throughout the world [1]. This symptom can result from almost any pathological condition affecting the auditory system including infections, hearing loss, cardiovascular disorders, metabolic disorders, or neoplasms [2]. Chronic tinnitus can also be caused by head trauma [3], numerous medications including quinine, salicylates, loop diuretics, aminoglycoside antibiotics, and some antineoplastic drugs [4]. The Physician's Desk Reference [5] lists dozens of medications that include tinnitus as a potential side effect. In most (but not all) cases, tinnitus resolves soon after patients stop taking the causative medication.

 This paper describes a case of chronic tinnitus that began after the patient received electroconvulsive therapy (ECT) for major depression. Numerous studies have shown that the severity of tinnitus is positively correlated with the self-rated severity of depression [6, 7]. That is, patients who rate their tinnitus as a severe problem tend to score higher on instruments such as the Beck Depression Inventory. However, depression was not the cause of tinnitus in these studies nor in the case report presented here. The patient in this report also had a long history of obsessive-compulsive disorder (OCD). A study by Folmer et al. [8] demonstrated that the severity of chronic tinnitus is positively correlated with a self-administered measure of obsessive-compulsiveness. While tinnitus might trigger or exacerbate such psychological conditions, the presence or worsening of depression or OCD do not initiate the perception of tinnitus. Also, tinnitus patients in general do not score significantly higher on measures of depression [7] or obsessive-compulsiveness [8] than people who do not perceive tinnitus, but the correlations between self-rated tinnitus severity and these psychiatric disorders are significant.

## 2. Case Report

The patient was a 43-year-old Caucasian female with a 27-year history of obsessive-compulsive disorder and major depression. Obsessiveness about her appearance began during her teen years when she experienced facial scarring from severe acne. In spite of ongoing treatment that included psychotherapy and at least eight different antidepressant medications (including several serotonin-specific reuptake inhibitors and tricyclics), the frequency and duration of depressive episodes increased during the last ten years. After two recent suicide attempts, the patient was scheduled to receive a series of ECT treatments.

 The patient underwent ECT procedures once per week for four weeks. ECT was administered according to the following parameters: Succinylcholine, glycopyrrolate, and methohexital were given intravenously prior to electrode placement. After the patient was under anesthesia, a stimulating electrode was placed on the right side of the scalp in the frontotemporal region. 0.9 A of stimulating current was delivered by a Thymatron ECT instrument (Somatics Inc., Lake Bluff, Illinois) on a setting of 40% (224 total 1 msec bidirectional pulses were delivered during 2.24 sec). 

**Figure 1:**
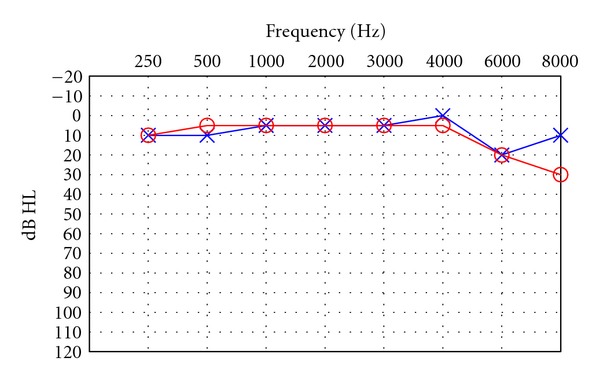
Pure-tone air conduction thresholds one day after ECT. ○: right ear thresholds; ×: left ear thresholds.

 Electroencephalographic (EEG) seizures were elicited within 15 seconds after stimulus presentation during all four procedures (seizure duration ranged between 30–45 sec). After recovering from anesthesia following each procedure, the patient complained of headaches, muscle pain, and amnesia. After the fourth ECT procedure, she also reported hearing a high-pitched ringing sound in her right ear. The day after this procedure, she went to an otolaryngologist and was given a hearing test. Audiometric results (pure tone air conduction thresholds) from this test are shown in Figure 1. These results indicate essentially normal hearing with a slight increase in threshold at 8000 Hz for the right ear. 

**Figure 2:**
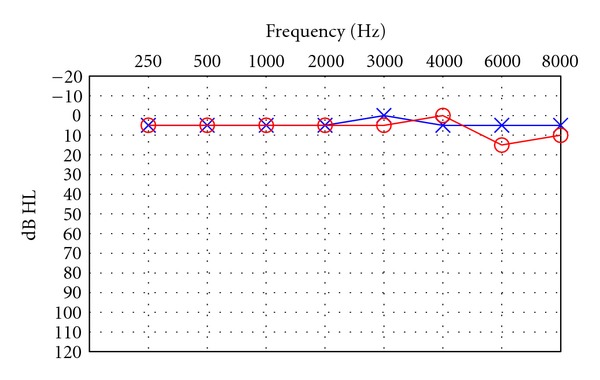
Pure-tone air conduction thresholds six weeks after ECT. ○: right ear thresholds; ×: left ear thresholds.

 Six weeks after the fourth ECT procedure, the patient was evaluated in the Tinnitus Clinic at Oregon Health & Science University (OHSU). In addition to tinnitus and depression, the patient reported that she was also experiencing anorexia, excessive weight loss, and insomnia. She reported that she was taking clonazepam, quetiapine, and citalopram, but none of these medications was particularly effective. Pure tone air conduction thresholds recorded during this appointment are shown in Figure 2. Compared to the audiogram recorded six weeks earlier, her thresholds at 6000 and 8000 Hz improved in both the left and right ears. However, the patient reported no noticeable reduction in tinnitus loudness. In fact, tinnitus was now perceived in both ears, but it was louder on the right side. She matched the sound of her tinnitus to a 4000 Hz tone at a loudness of 10 dB sensation level (SL) in the right ear and 5 dB SL in the left ear. The following recommendations were made to her: (1) That she uses pleasant sounds to give her relief from tinnitus whenever possible. To facilitate this acoustic therapy, she purchased two in-the-ear sound generating devices (General Hearing Instruments, New Orleans, LA), a Sound Pillow (Phoenix Productions & Promotional Products, San Antonio, TX—this pillow is embedded with two flat and flexible speakers that can be connected to any electronic sound producing device; the patient can then play any sort of pleasing or comforting music or sounds to help with sleep), and a CD with waterfall and nature sounds. (2) That she talks with her physicians about ways to improve her diet/appetite and to supplement her meals with multivitamins/minerals or nutritional beverages. (3) That she talks with her physicians about using alprazolam to reduce anxiety and the loudness of her tinnitus. She was given a reprint of a study conducted in the OHSU Tinnitus Clinic that used alprazolam for this purpose [9]. (4) That she talks with her physicians about alternative treatments for insomnia. 

 After leaving the OHSU Tinnitus Clinic, the patient was transported to a psychiatric care facility for long-term evaluation and treatment. She continued to call the clinic on a regular basis (as she did before scheduling her appointment) to ask us if the tinnitus will ever stop. Our answer, which was always unsatisfactory to this patient, was that we cannot say for sure, but the fact that her hearing recovered might be a positive indicator that her perception of tinnitus will eventually decrease. The acoustical therapy devices she purchased can facilitate this process. Two years after her Tinnitus Clinic appointment, the patient's tinnitus decreased to the point where it was barely perceptible, even in quiet environments.

## 3. Discussion

One could argue that this patient's tinnitus was caused by the medications that were used to prepare her for ECT. In fact, all of these medications have the potential for producing the side effect of tinnitus that is usually temporary. However, the patient did not experience tinnitus following the first three ECT procedures which used the same medications in the same dosages. Citalopram, clonazepam, and quetiapine that the patient had been taking can also trigger tinnitus perceptions in some people. However, the patient had been taking these medications for several weeks or months prior to her fourth ECT and had not previously reported this side effect. Because the patient reported that she heard tinnitus immediately after she recovered from anesthesia, it is likely that her fourth ECT procedure produced this symptom. 

 The most common cause of tinnitus is damage to the cochlea that also results in hearing loss [2]. It is possible that the ECT procedures damaged the patient's cochlea directly or caused changes in central auditory pathways/functions that resulted in tinnitus and reduced hearing sensitivity. A report by Shepherd et al. [10] provides some evidence for this theory. These authors describe cochlear damage and hearing loss in a cat that inadvertently received a short burst of direct current (DC) stimulation. Also, two published case studies reported tinnitus perception in humans that was coincident with epileptiform activity [11, 12]. 

 Three additional facts lend credence to the theory that ECT caused this patient's tinnitus to start (1) the stimulating electrode was placed in the right temporal region of her scalp; (2) audiometric testing of the patient the day after her fourth ECT treatment showed reduced sensitivity for 8000 Hz tones in her right ear; (3) the patient originally perceived tinnitus in the right ear only. Even though the hearing in her right ear improved six weeks after the last ECT, the patient's perception of tinnitus remained constant during this period. This observation is consistent with the model of tinnitus generation proposed by Lenarz et al. [13] who stated that “the induction of tinnitus usually involves pathologies of peripheral auditory structures” and “the manifestation and maintenance of tinnitus involves central auditory structures which are not necessarily impaired.” 

 Why didn't this patient experience tinnitus after her first three ECT procedures? It is possible that these procedures were causing subtle changes to occur in peripheral and/or central auditory structures, and that the fourth ECT session was the trigger for tinnitus to begin. The unique organization of this patient's central nervous and auditory systems combined with her particular pharmacological history might have predisposed her to developing tinnitus. Two published case studies reported that tinnitus and depression were successfully treated by ECT in the same patients [14, 15]. However, Charles [16] reported that existing tinnitus worsened in a depressed patient after he received ECT, although the patient's depression improved. Regarding tinnitus worsening after ECT, Charles [16] asked, “I wonder if any other psychiatrists have encountered such a side-effect?” The fact that we found no other published reports of ECT causing the symptom of tinnitus indicates that it is probably a rare side effect. We, too, are interested in hearing from other clinicians whose patients reported either acute or chronic tinnitus associated with ECT.
